# Annual Variation in Flowering Phenology, Pollination, Mating System, and Pollen Yield in Two Natural Populations of *Schima wallichii* (DC.) Korth

**DOI:** 10.1155/2013/350157

**Published:** 2013-12-31

**Authors:** Vinod Prasad Khanduri, C. M. Sharma, K. S. Kumar, S. K. Ghildiyal

**Affiliations:** ^1^Department of Forestry, Mizoram University, Aizawl, Mizoram, India; ^2^Department of Forestry, Uttarakhand University of Horticulture and Forestry, Hill Campus, Ranichauri, Tehri Garhwal, Uttarakhand, India; ^3^Department of Botany, HNB Garhwal University, Srinagar Garhwal, Uttarakhand, India

## Abstract

*Background*. *Schima wallichii* is a highly valuable tree of tropical forest in north-east Himalaya region that grows naturally in a wide range of altitudes between 750 and 2400 m asl with varying environments. Flowering phenology of tropical tree species at population level is generally ignored and therefore a detailed knowledge of flowering and fruiting patterns of important multipurpose tree species is critical to the successful management of forest genetic resources. *Materials and Methods*. The study was conducted at two different altitudes (i.e., 750 m and 900 m asl) in the tropical semideciduous forest of north-east Himalaya. The floral phenology including flowering synchrony in the populations, anthesis, anther dehiscence, stigma receptivity, pollinators visitation frequency, and mating system including index of self-incompatibility were worked out in *Schima wallichii* according to the ear-marked standard methods given by various scientists for each parameter. *Results*. The flowering period in *Schima wallichii* varied from 33 to 42 days with mean synchrony of 0.54 to 0.68 between the populations. The stigma was receptive up to 2.5 days only and showed slightly protandrous type of dichogamy. Average pollen production ranged between 6.90 × 10^7^ pollen per tree in 2007 and 15.49 × 10^8^ pollen per tree in 2011. A three-year masting cycle was noticed in this species. The frequency of visitation of honey bees was fairly high (5.2 ± 1.12 visits/flower/hour) as compared to other pollinators. The hand pollination revealed maximum fruit (74.2 ± 5.72%) and seed (70.8 ± 7.46%) settings. *Conclusions*. The variation in flowering phenology and pollen yield individually and annually along with temporal separation in anther dehiscence and pollinator's visitation cause pollen limited reproduction, which ultimately influences the reproductive success in *Schima wallichii*.

## 1. Introduction

Synchronous and asynchronous flowerings within the plant populations determine the level of out-crossing and selfing, which ultimately influence the reproductive success of the plants [1, 2]. During the overlap of blooming periods, plant species utilizes the same pollinators, which results in competition for pollination [3]. Pollination makes first male-female interactions and is fulfilled by abiotic and biotic factors in nature. For the entomophilous species pollinators are important selective agents acclimatized to flower form and size [4]. The overall visitation rate of flowers [5] and subsequent seed set [6] are primarily based on the nature and size of the flowers [7, 8]. The floral display and number of flowers opened daily represent the potential for visit of pollinators [9] and pollination success [10]. However, the open floral architecture together with provision for abundant nectar and pollen ensures the visit of diverse range of insect pollinators [11]. Therefore, study of the breeding systems of any species requires assessment of floral display, which is a prerequisite for knowing the pollinator behaviour and outcrossing rate [12].

Individuals of a plant species with hermaphrodite flowers may have chance of autogamous self-pollination, which leads to maximum seed setting but minimum genetic variation in their offspring. Gradually the flowers however evolve dichogamy separation in maturation time of stigma and pollen to avoid autogamous self-pollination and herkogamy (physical variation) as was also noticed [13, 14]. Geitonogamy (interfloral pollen transfer within and between inflorescences) and the level of flowering synchrony within the plant also influence the pollination. However the most effective antiselfing mechanism in angiosperms is self-incompatibility. Most of the self-incompatible angiosperm species are woody plants [15, 16]. Self-incompatibility mechanisms of the species in a population can be monitored by studying the fruit or seed production following self- and cross-pollination, and the degree of self-incompatibility can be quantified by using an index (ISI) of self-incompatibility [16].

To achieve the breeding goal, a tree breeder needs to formulate a breeding strategy or tree improvement plan that must require a complete review of species in terms of silviculture, biology, and genetics. Flowering phenology of tropical tree species at population level has not been given the required heed thus far and therefore a detailed knowledge of flowering phenology and fruiting pattern is critical for successful management of forest genetic resources [17, 18]. It has been however reported that at population level there are considerable year-to-year quantitative variations in flowering and fruiting [17, 19]. This paper emphasizes the complete reproductive biology of *S. wallichii*, which can be used for provenance selection, Development and establishment of seedling seed orchards (SSOs), seed production areas, and further genetic testing of this valuable timber yielding species in the semideciduous forests of eastern Himalaya and other parts of the globe, having similar edaphoclimatic conditions.

## 2. Material and Methods

### 2.1. Study Site

**Figure 1:**
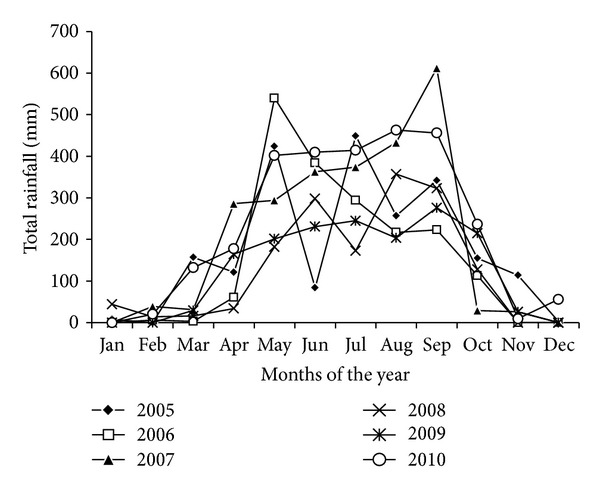
Rainfall data of the study area over the past six years.

The study was conducted in two different locations of tropical forest in north-east Himalaya, namely, (i) Tanhril (inside the Mizoram University Campus) located at 750 m asl. (23°44′13′′N latitudes and 92°39′38′′E longitudes) and (ii) Bethlehem Vengthlang located at 900 m asl. (23°43′30′′N latitudes and 92°43′54′′E longitudes). Both the study sites were situated in the district Aizawl, Mizoram (the north eastern hill region: NEH), India. The *Schima wallichii* was dominant species in the forest of Tanhril in association with *Callicarpa arborea*, *Erythrina stricta*, and *Sterculia* spp. The forest of Bethlehem Vengthlang was equally dominated by *Anthocephalus cadamba*, *Gmelina arborea*, *Schima wallichii*, *Erythrina stricta*, and* Castanopsis *spp. The regional climate of the area is seasonal with 80% of rains (total average of six years is 2063.18 ± 219.80 mm) concentrated in the period from May to September (Figure 1). The variation in total annual rainfall is very high which ranged between 1569 mm (in year 2008) and 2777.3 mm (in year 2010).

### 2.2. General Characteristics of *Schima wallichii*

*Schima* is a monotypic, polymorphic genus belonging to the family Theaceae that has a single species, *S. wallichii*. (DC.) Korth. [20]. It is an ever-green tree native to warm temperate and tropical regions of southern and southeastern Asia. *Schima *is mostly used for fuel, poles, timber, and occasionally for fodder [21]. The tree attains a height up to 35 m with dense crown and stem diameter up to 125 cm. Leaves elliptic-oblong or elliptic-lanceolate, 6–15 × 4–6 cm in size; flowers white, 2.5–4 cm across, scented, axillary solitary or paired. It has a broad distribution occurring from northeastern India through southern China, Thailand, peninsular Malaysia, Sumatra, Java, Borneo, and the Philippines and adaptive to wide ranging of environments including dry sites, infertile soils, and clay soils [22]. *S. wallichii* is fire resistant [23], moderately light demander, often occurs gregariously in primary lowland to montane forests, scrublands and grasslands but is particularly common in disturbed and secondary forests [24].

### 2.3. Floral Phenology

Five phenological parameters were derived from the flowering data: (i) onset (date of first flower in anthesis); (ii) duration (date of first and last flower); (iii) mean flowering date (peak of flowering): the average of the census dates during which that individual was flowering, with each census date valued by the number of flowers in that period; sensu [25]; (iv) mean flowering amplitude (number of flowers produced per unit time, sensu [19]; and (v) synchrony (flowering overlap among individuals). All these variables of flowering phenology except synchrony were derived for both individual plants and the population as a whole. Ten individuals in each population were chosen randomly and marked with yellow paint and the same individuals were used for sampling every year. The method used for floral phenology was closely followed as of [26].

Flowering synchrony within an individual plant was the degree to which blooming period of the plant overlapped the blooming period of all the other plants within the population. Synchrony was calculated using the method of [27], modified by Primack [28]. For each individual, the number of weeks when the flowering overlapped that of other individuals was estimated. The index of synchrony (*X*) for an individual plant (/) was estimated as

(1) $$\begin{array}{c} X_{i}=\left(\frac{1}{n-1}\right)\left(\frac{1}{f_{i}}\right)\sum_{J=i}^{n}e_{j\neq i}, \end{array}$$

where *e*_*j*_ is the number of weeks individual *i* and *j* overlapped their flowering; *f*_*i*_ is the total number of weeks individual *i* was in flower, and *n* is the number of individuals in the sample. *X* varies from 1 (plant flowering overlaps with that of all other individuals) to 0 (no overlap with any other individuals).

### 2.4. Anthesis, Anther Dehiscence, and Stigma Receptivity

Flower development was monitored during the flowering season on the selected ten individuals. On each tree, twenty flower buds of similar stage were marked and subsequently observed for anthesis, anther dehiscence, and stigma receptivity after every 2 h interval of the day from dawn (0500 h) to dust (1800 h). Anthesis (by definition is the opening of flowers to display the reproductive sex organs) was observed on the marked flowers on each tree to know the pattern of anthesis and time taken by a flower for complete opening. Anther dehiscence was assessed at the time of anthesis by using a hand lens (×20) to know the time differences between anthesis and anther dehiscence, and anther dehiscence and stigma receptivity. Receptivity of stigmas was assessed after anthesis till the receptivity ended. It was tested after every two hour interval during the entire day length. The receptivity was tested through peroxidase activity, using a solution of 3% hydrogen peroxide [29]. Pearson correlation was used to assess the relationship between the climatic factors (temperature and humidity) with anthesis, anther dehiscence, and stigma receptivity.

### 2.5. Flower and Pollen Production

Flower production was estimated on ten trees selected and marked randomly on each location and the same trees have been monitored/sampled for each successive year. For the estimation of production of flowers per tree, first the total number of flower bearing branches per tree were counted and then the number of flower bearing boughs (inflorescences) per branch was estimated on five randomly chosen branches (in case of the trees where the total number of flowering branches was less than five all branches were considered) on the tree crown. The number of flowers per bough (or inflorescence) was counted on twenty different boughs selected randomly on the crown of each individual. Further, twenty-five flowers from every individual were harvested and the number of stamens was counted manually. The other floral characteristics, namely, the number of sepals, petals, flower diameter, style length, and ovule numbers were measured from the harvested flowers. The ovule number was directly obtained from dissection of ovaries under a stereoscopic microscope. The total number of flowers per tree was calculated by multiplying the number of flower per boughs by the number of flowered boughs per branch by the number of flowered branches per tree.

In order to estimate the production of pollen grains per flower and per tree, first the number of pollen grains per anther was counted from ten randomly selected flower buds in each individual. Five anthers from each flower, total fifty anthers (10 × 5 = 50) per tree, were used to estimate the number of pollen grains per anther. The average number of pollen grains from fifty anthers was used as a value for the number of pollen grains/anthers. One anther from each flower was taken and transferred to 1 cc of 50% glycerin in a test tube and macerated with a glass rod to suspend the pollen grains uniformly. Further, a sample of known volume, that is, 10 *μ*L was taken and placed in a microscopic slide and the pollen grains were counted under the binocular microscope [30, 31]. In order to reduce the chance of error, the number of pollen grains in an anther was also counted by placing the anther directly on the microscopic slides, crushed with glass rod, and a small drop of 50% glycerin was added to disperse the pollen grains equally to some fixed area on the slide and counted under microscope. The average of both the methods was used (*n* = 25 + 25 = 50) for the number of pollen grains per anther. For knowing the production of pollen grains per tree, the number of stamens per tree was calculated by multiplying the average number of stamens per flowers by the number of flower per tree. The resultant value was finally multiplied by the average number of pollen grains per anther.

The estimation of seed production per tree was done in the similar fashion as was described for flower production per tree. The total number of seeds per tree was calculated by multiplying the average number of seeds per fruit (*n* = 50) by the total number of fruits per tree. Seed production per fruit was estimated on 50 fruits per individual tree harvested randomly from different parts of the crown. Different parameters, that is, pollen grains per anther, the number of flowers per tree, pollen grains per tree, and seed number per tree, were statistically analyzed for analysis of variance. Pearson correlation was performed to observe the relationship between tree height versus production of pollen and seeds, pollen production versus seed production per individual, and rainfall versus pollen and seed production.

### 2.6. Flower Visitors and Pollination

To determine the pollinators and their visitation rates, counting of the visiting insect species and their frequency was done on all the ten chosen trees across all the years. The study period covered the fifteen days of peak flowering season after every alternate day in each location. The observations were made on ten randomly chosen boughs (inflorescences) per tree and recorded for the number of opened flowers. For each visit the number of visited flowers, the duration of each visit, contact with the reproductive parts, and interactions with other visitors were recorded. In each population the trees were observed over the course of the whole day length between 0500 h and 1700 h in 6 observation blocks, each starting at the 2-hour interval, that is, between 0500–0700, 0700–0900, 0900–1100, and so forth. The sustainable distribution of each observation block was done over the trees within the population during the course of the study so that every tree is monitored in each observation block. The frequency of pollinators was assessed in terms of visits/flower/hour. Honey bees, Carpenter bees, and butterflies were the main pollinators recorded for this species and observations were recorded only on these three types of pollinators. Frequency of visit was considered as a measurement of pollination efficiency [32].

### 2.7. Assessment of Mating System

The assessment of breeding system involved ten trees in each population during the year 2008, when profuse flowering was occurred. For this purpose the following treatments were performed: (i) natural pollination (flowers were not manipulated); (ii) autogamous self-pollination (buds were bagged by fine net throughout their flowering period); (iii) hand self-pollination (bagged flowers were hand pollinated with their own pollen); (iv) hand cross-pollination (emasculated bagged flowers were pollinated with pollen from another tree); and (v) apomixis (anthers and stigma of buds were clipped). All the aforesaid treatments were done on each of the ten individuals in a population. This was done by selecting three boughs for each treatment per tree and a total of fifteen boughs per tree for all treatments. In each population every treatment therefore contained thirty boughs. The flower numbers per boughs were 18 to 26. To compare fruit setting among hand-selfed and hand cross-treatments, and among open pollinated and hand-cross-treatments, Chi-square analysis was performed [33] to know the pattern of variability between these treatments. An indirect measure of self-incompatibility was obtained by dividing the average fruit set after hand self-pollination by the average fruit set after hand cross-pollination [34, 35]. The resultant value of index reflected the possibilities as (i) >1 = self-compatible; (ii) >0.2 and <1 = partially self-incompatible; (iii) <0.2 = mostly self-incompatible; and (iv) 0.0 = completely self-incompatible [34].

## 3. Results

### 3.1. Floral Phenology

During the six years of study, it was observed that the time of flowering was almost the same; however, there was little fluctuation in the time of onset and flowering period, as the date of onset was 19th March and 25th March in 2006 and 2008, respectively. Nevertheless, during the years 2007 and 2010 it was 7th and 2nd April, respectively. The flowering period actually was varied from 33 to 42 days with the mean synchrony of 0.54 to 0.68 (Table 1). Time between onset and peak flowering varied from 3 weeks (2007 and 2010) to 4 weeks (2006, 2008, and 2011). Flowering amplitude was the highest in the mass flowering years (i.e., 2008 and 2011), followed by nearly equal production in rest of the years. On an average the date of start of anthesis was 15 days after initiation of floral buds. The total duration of flowering period in a population was about one month. The flowering was partially synchronized within a population and was simultaneous within the individual. It was also observed that the trees which flowered earlier in one year followed the similar trend in successive years.

### 3.2. Anthesis, Anther Dehiscence, and Stigma Receptivity

**Figure 2:**
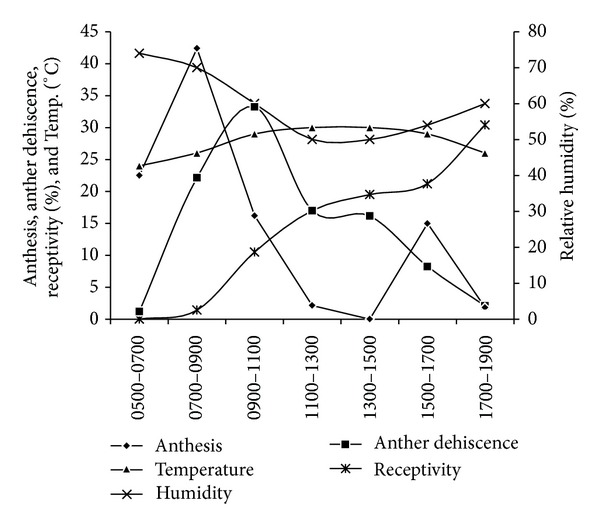
Pattern of anthesis, anther dehiscence and stigma receptivity in relation to the time of the day in *S. wallichii. *

Anthesis in flowers started from 0600 h in the morning and reached to its maximum between 0700 and 0900 hours of the day. As the day progressed, the level of temperature increased and relative humidity (RH) decreased at noon, the progress of anthesis decreased and gone down to its minimum level between 1100 and 1300 h of the day (Figure 2). Similarly, the flower openings were again recorded during the afternoon hours between 1500 and 1700 h, which might be due to the decrease in temperature and increase in RH levels. A flower took 2 h for complete opening. The correlation analysis also verified this trend as there was significant negative (*r* = −0.5345; *P* = 0.122) and positive (*r* = 0.7692; *P* = 0.024) relationship of anthesis with temperature and humidity, respectively. The anthesis was not recorded during night time. This shows that light is also equally important for the process of anthesis in *S. wallichii*.

Anther dehiscence started one hour after anthesis, that is, 0700 h of the day and followed a diurnal pattern (Figure 2). Both the significant effects, that is, (i) prevention of anther dehiscence at reduced temperature and (ii) inhibition by high humidity during morning and evening hours of the day, along with normal sequence of changes were observed when the temperature and humidity were returned to a more standard value, at mid-day time. Therefore the correlation was significantly positive (*r* = 0.5117; *P* = 0.100) and negative (*r* = −0.1759; *P* = 0.214) with temperature and humidity, respectively.

The receptivity of stigma started from 1000 h of the day (after 4 h of anther dehiscence). This indicates slightly protandrous type of dichogamy in *S. wallichii*. The stigma remained receptive up to 2.5 days. Receptivity proclaimed both positive and negative relationship with temperature (*r* = 0.4471; *P* = 0.310) and humidity (*r* = −0.7236; *P* = 0.014) respectively, which may have positive consequence for manipulating the female receptivity. Despite the peroxidase test, the receptivity of stigma can be judged by the colour change, which may act as an identification mark for knowing the receptivity [30]. It was observed that the stigma was off-white in colour in the prereceptive stage whereas it turned to greenish grey the receptive stage.

### 3.3. Flower and Pollen Production

The results of the production variables (branches that produce flower per tree, number of boughs (inflorescence) per branch, flower per bough, stamen per flower, pollen grains per tree, fruits per tree, and seeds per tree) have shown great variation between the years. During the course of six-year study (between 2006 and 2011), there were two good production years (2008 and 2011), three poor production years (2006, 2007, and 2010), and a year of complete crop failure (2009). Good production years (2008 and 2011) were considered as mast production years because flowering was profuse (when all individuals in the population borne flowers) in both populations. It was interesting to note that the mass flowering was followed by complete failure of flowering in both populations in the next year 2009. The mass flowering was repeated in the year 2011, clearly depicting three-year mast seeding or flowering cycle in *S. wallichii*. In the poor production years the number of flowered boughs (inflorescences) per branch was 8.67 (average of three years) which in the mast years was recorded as 20.8 boughs per branch. Similarly, the flowers number per bough varied substantially in the mast and poor production years. The mast production years produced 40% more flowers per bough than the poor production years. It was also observed that, during the poor flowering years, some individuals in a population did not bear flower. The stamen number per flower was equally variable in different years. A flower produced 139 to 156 stamens per flower. The range of variation was narrow in terms of stamens per flower and pollen grains per flower. However, poor production years amounted slightly higher number of anthers (4% more) and pollen grains (11% more) per flower than the mast production years. There was a substantial difference in the production of pollen grains per tree between mast and poor production years. The average number of pollen grains that a tree produced in the mast years was 15.49 × 10^8^ (2011), which in the poor production years was 6.90 × 10^7^ (2007). The fruit and seed settings were fairly high in the mast production years and consequently the number of seeds per tree was 96% more than the poor production years (Table 2).

There were significant differences in the production units, namely, number of flowers (*F* = 41.05, *P* = 0.0012), pollen grains (*F* = 61.74, *P* = 0.0001), and seed number per tree (*F* = 72.46, *P* = 0.0001) in different years. The analysis of production of pollen grains per anther proclaimed a nonsignificant year effect (*F* = 18.72, *P* = 0.243). Tree height was found to be strongly positively correlated (*r* = 0.8746; *P* < 0.0001) with the number of flowers and fruits (*r* = 0.5432; *P* < 0.01) produced in all five flowering years, which leads to demonstrate the effect of tree size on fecundity. There was strong relationship between pollen and seed production (*r* = 0.7485, *P* < 0.0001) in all the studied years, which clearly addressed a lag between pollen and seed production. A strong relationship was observed between the preceding years rainfall and both pollen (*r* = 0.9331, *P* < 0.0001) and seed (*r* = 0.8697, *P* < 0.0001) production. The total annual rainfall was maximum during the years 2007 (2487 mm) and 2010 (2777.3 mm), as compared to other years, that is, 2005 (2113 mm), 2006 (1841 mm), 2008 (1569 mm), and 2009 (1591.8 mm). The fruits or capsules of *S. wallichii* become mature in 9-10 months after pollination and the colour of fruits gradually turns to green and then to light brown. This is the best collection time of fruits before they crack open and seeds are dispersed by wind. The dehisced fruits do not fall down and remain attached to the branches for one year.

### 3.4. Pollinator Visitation

**Figure 3:**
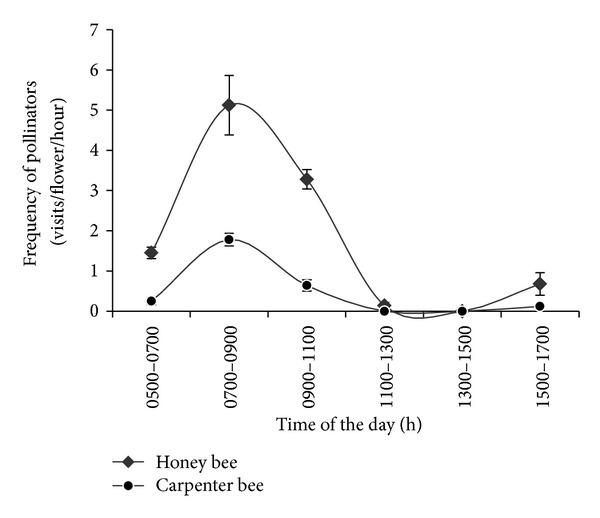
Frequency of pollinators' visitations in respect to the time of the day.

**Figure 4:**
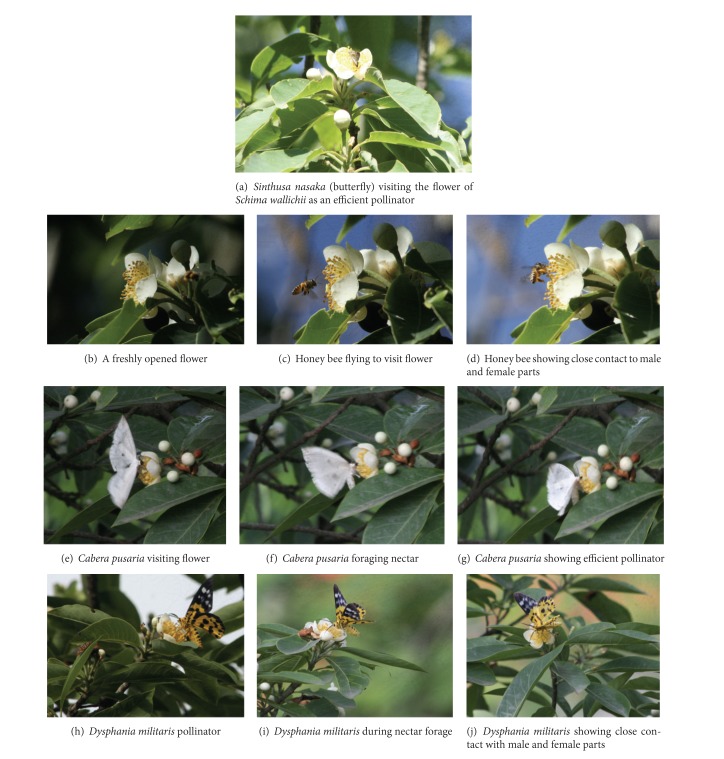
Different pollinators of *Schima wallichii*.

Honey bees (*Apis *sp.) and carpenter bees (*Xylocopa *sp.) are the most effective pollinators of *S. wallichii*. However, the frequency of honey bees was fairly high (5.2 ± 1.12 visits/flower/hour) as compared to carpenter bees (1.7 ± 1.12 visits/flower/hour) during peak visiting hours (between 0700 and 1100 h). Moreover, the pollinator visitation was also coincided with the pattern of anthesis (Figure 3). Furthermore, the average time that a single pollinator spends in the flower was 3.2 ± 0.16 minutes (for honey bee). It was also observed that the pollinator remains in close contact with male and female reproductive organs during this period (Figures 4(b)–4(d)), which for carpenter bee was a 0.32 ± 0.09 minute. Three butterfly species, namely, (i) *Sinthusa nasaka* (Figure 4(a)), (ii) *Cabera pusaria* (Figures 4(e)–4(g)), and (iii) *Dysphania militaris* (Figures 1(h)–1(j)) were also recorded as the effective pollinators in *S. wallichii* with the maximum visitation of 2.42 ± 0.38 visits/flower/hour for *Dysphania militaris*.

### 3.5. Mating System Studies

The results of the pollination experiment are summarized in Table 3. Flowers were also used to test for apomixis which did not set fruit. There are significant differences between hand selfed and hand cross treatments (*x* ^2^ = 25.25, *P* < 0.0001) and between the open pollinated and hand cross treatments (*x* ^2^ = 15.82, *P* < 0.001). Hand cross treatment was most successful than hand self-treatment and open pollination. The results of hand cross-pollination revealed that maximum fruit (74.2 ± 5.72) and seed (70.8 ± 7.46) se are followed by open pollination (65.4 ± 6.42 and 56.6 ± 8.54, resp.). The autogamous self-pollination proclaimed the least fruit (15 ± 1.14) and seed (21.4 ± 2.32) settings. The index of self incompatibility (ISI) value was estimated as 0.52 ± 0.12, which showed that the *S. wallichii* is a partially self-incompatible species.

## 4. Discussion

### 4.1. Flowering Phenology

The variation in flowering phenology was observed from year to year within each population. There was synchrony in blooming within the populations. Synchronization in flowering within a population is of prime importance for successful seed setting and maximum outcrossing level, which will determine the partitioning of genetic variation in the population [36]. Synchrony among individuals increases cross-pollination via attraction of pollinators [27]. Pollinator visitation frequency and plant reproduction increase with flowering synchrony [32]. Trees that flower early tend to have shorter flowering period. The overall reproductive period in *S. wallichii* was 36 days in an individual and 49 days in the entire population, which is less than that found in *Tectota grandis* [37], whereas comparable to other conifers [38, 39]. The year to year variation in reproductive phenology in *S. wallichii* varies by about 10 days which is lower than that reported for *T. grandis* [37] and *Pinus contorta* [40].

### 4.2. Anthesis, Anther Dehiscence, and Stigma Receptivity

Anthesis was more temperature dependent, and anther dehiscence was strongly influenced by relative humidity. Anthesis and anther dehiscence are very sensitive to the fluctuation of weather factors, mainly temperature and humidity. Similarly, both factors had the influence on receptivity of female. This would be utilized in the seed orchard to extend the receptivity period by manipulating the temperature and humidity *via* overhead water cooling treatment, as has been practiced in a Douglas-fir seed orchard [41]. There is a difference in anther dehiscence and stigma receptivity. The receptivity starts 4 hour after dehiscence, clearly suggesting the protandrous dichogamy in *S. wallichii*. This has important consequences for promoting outcrossing in this species, as there is synchrony in blooming with in the population.

### 4.3. Flower and Pollen Production

Pollination of the flowers of individual tree may be influenced by its own pattern of flower production and/or by its synchrony with conspecifics [27]. Tree height had significant positive relation with the production of flowers and fruits, which has translated the effect of tree size on fecundity. Larger trees produced more fruits and seeds than smaller trees and are at a reproductive advantage in the population. As a consequence the largest plants in a population are usually the most fecund. Similar results were also reported for other species [25, 26].

The analysis of pollen and seed production per tree revealed strong temporal variation in *S. wallichii*. The total crop failure in 2009 suggested that the species played a significant role in the phenomenon of masting or mast seeding. The mass flowering was repeated in the year 2011, showing a three-year cycle of mast seeding. Pollen production is an important contributor to masting [42], as a result a strong relationship between pollen and seed production was observed in *S. wallichii*. This was also reported for oaks [43] and *Ponderosa pine* [42]. The synchrony of flowering among individuals as well as lag between pollen and seed production is very important for the good seed crop year [44].

The interannual climatic variation is associated with the variation in pollen and seed production, because a significant positive relationship for the preceding years rainfall was observed for both variables. Climate has been considered as an important resource to enhance or limit patterns of reproduction [45]. The high annual rainfall before mass flowering may be interpreted as an environmental cue for profuse flowering in *S. wallichii*, as precipitation is one of the principal factors influencing the high productivity of trees [46]. In Mediterranean ecosystems, water availability plays an important role in determining phenological development [47]. Seasonal variations in rainfall have been reported as proximate cause triggering flowering in tropical species [48]. However, pollen yield is regarded for regulating masting as the physiological cost of reproduction [49] in addition to the climate, as a cue for masting [50].

### 4.4. Pollination and Visitation

Decline in the frequency of pollinator's visit after 1100 h of day (Figure 3) reduced the quantity and quality of the pollen deposited on stigmas, as the stigma becomes receptive mostly after 1100 h of the day (Figure 2), which may limit fruit and/or seed production [51]. On the other hand this has a strong positive consequence for promoting cross-pollination (as the seed set from experimental self-pollination is low as compared to cross-pollination). The extent of contact between a pollinator and a flower's anther seems largely to determine the rate of pollen removal. The duration of visit certainly increased the removal of pollen [52]. This would also depend on the anther dehiscence of a flower. Once the dehiscence started after anthesis, the entire flower dehisced within four hours. The dehiscence was diurnal showing that the pollen inside the flowers was available throughout the day. However, the frequency of visitation of the pollinators was higher in forenoon hours which dictate the time separation for presenting pollen grains. As a result the chances of pollen limited reproduction are more within the population [53]. Furthermore, a four-hour difference in the anther dehiscence and stigma receptivity in terms of protandry was also responsible for pollen limitation, although promoting outcrossing in *S. wallichii*.

### 4.5. Mating System

The number of ovules that develop into seeds was significantly very low in self-pollinated as compared to hand cross-pollinated and open-pollinated systems resulting in low seed/ovule ratios in self-fruits (capsules) only. Fruit setting followed by open and hand cross-pollination suggested that the all ovules in ovaries of *S. wallichii* were potentially fertile. Furthermore, the seed production was pollen limited even if the trees were producing copious number of stamen and pollen grains with comparatively high frequency of pollinator's visit. The pollen limited reproduction was mainly due to the time differences in anther dehiscence and frequency of pollinator visitation. Furthermore, results of this study on the floral biology and the breeding system of *Schima wallichii* indicated that the species' reproductive potential was dependent on the synchrony in flowering phenology and pollen limitation which limit the production of selfed seeds to maintain the level of outcrossing in populations.

Floral phenological differences and pollen productivity among trees within stands influenced the mating success, as reproductive success increased with increasing pollen yield and with increasing flowering overlap in mass production years. Furthermore, tree density in a population with high density of flowering influenced the mating system by increasing the level of outcrossing among individuals [54]. This would be true to *S. wallichii*, because, in the mass flowering years, the fruit set was high as compared to poor flowering years. High flowering density with flowering overlap increased the pollination efficiency to the tropical tree species whose pollination was mediated by insects [55].

Information on reproductive mechanism of natural population of any species provides base line data for conducting the successful tree improvement programme in future. For example *S. wallichii* produce mass flowering after 3-year interval, which would be a key information to those working on provenance testing and selection of seed production areas. Furthermore, the results could be utilized for the planning and management of mating system strategies in natural stands and management of seed orchards of *S. wallichii*. Before conducting any improvement/breeding programme in any species the main objective would be to select the individual(s) with traits that have aggregate breeding value [56]. However, in *Schima wallichii*, the important breeding trait would be that the natural population has variation in flowering phenology, pollen yield which may affect pollinator visitation. Thus the resultant pollen limitation may subsequently influence the reproductive success in this species.

## Figures and Tables

**Table 1:** Phenology data at the plant and population levels. For plant data, values shown are the mean of all plant values ± standard deviation. For population data, values given represent whole population (i.e., the onset is the date of the first flower on the first plant in the population). Dates are given in the calendar date.

Observed variables	2006	2007	2008	2010	2011
Onset (mean plant values)	26th March	14th April	31st March	9th April	1st April
Onset (population)	19th March	7th April	25th March	2nd April	28th March
Flowering duration (plant)	38 d ± 5	42 d ± 6	36 d ± 4	33 d ± 5	34 d ± 4
Flowering duration (population)	54 d	48 d	52 d	42 d	48 d
Average date of blooming (plants)	21 April	1st May	22 April	29 April	25 April
Average date of blooming (population)	17 April	22 April	16 April	21 April	18 April
Synchrony (plants)	0.64 ± 0.12	0.60 ± 0.10	0.68 ± 0.12	0.54 ± 0.10	0.64 ± 0.16
Amplitude (flowers/branch/day): plants	0.52 ± 0.16	0.46 ± 0.12	0.48 ± 0.10	0.42 ± 0.14	0.46 ± 0.14
Amplitude (flowers/branch/day): population	0.32 ± 0.10	0.30 ± 0.10	0.36 ± 0.12	0.32 ± 0.12	0.36 ± 0.10

**Table 2:** Annual production of flower, pollen, and seeds per tree in *S. wallichii*.

Observed variables	2006	2007	2008	2010	2011	Grand average
Average diameter (cm)	28 ± 2.14	—	—	28.8 ± 2.20	—	28.4
Average height (m)	12 ± 1.10	—	—	13 ± 1.18	—	12.5
Flowering bearing branches/tree	04 ± 0.12	02 ± 0.08^(−50%)^	10 ± 1.12^(80%)^	03 ± 0.10^(−70%)^	10.6 ± 1.25^(72%)^	5.9
Flowering bearing bough/branch	10 ± 1.14	8 ± 0.98^(−20%)^	21 ± 3.18^(62%)^	8 ± 0.42^(−62%)^	20.6 ± 1.08^(61%)^	13.5
Flowers/bough	14 ± 1.62	15 ± 2.14^(12.5%)^	24 ± 1.98^(33%)^	14 ± 1.68^(−42%)^	25.2 ± 2.15^(44%)^	18.6
Stamens/flower	156 ± 20.14	146 ± 22.16^(−6%)^	139 ± 30.26^(−5%)^	142 ± 19.16^(2%)^	145.6 ± 20.92^(−2%)^	145.7
Pollen grains/anther	1946 ± 415.13	1874 ± 540.42^(−4%)^	1796 ± 804.12^(4%)^	1901 ± 215.14^(−6%)^	1796 ± 465.05^(6%)^	1862.6
Pollen grains/flower	308690 ± 13630.70	270604 ± 10230.40^(12%)^	245444 ± 10014.2^(9%)^	271390 ± 11842.6^(−11%)^	260977 ± 9961.1^(4%)^	271421
Pollen grains/tree	16.94 × 10^7^ ± 7.9 × 10^6^	6.90 × 10^7^ ± 1.2 × 10^6(59%)^	13.38 × 10^8^ ± 11.78 × 10^7(−95%)^	9.13 × 10^7^ ± 1.79 × 10^6(93%)^	15.49 × 10^8^ ± 3.94 × 10^7(−94%)^	6.43 × 10^8^
Fruit setting (%)	49 ± 6.12	28 ± 2.14^(43%)^	68 ± 6.18^(−59%)^	36 ± 6.74^(47%)^	62 ± 4.86^(42%)^	48.6
Average seeds/tree	6640 ± 44.25	1712 ± 123.62^(74%)^	92764 ± 892.6^(−98%)^	2658 ± 74.8^(97%)^	78268 ± 642.4^(−96%)^	36408

Superscript figures in parentheses shows percent difference between two years; namely 2006 and 2007, 2007 and 2008, 2008 and 2010, and 2010 and 2011; dashes (—) indicating lack of data.

**Table 3:** Fruit and seed setting following mating system experiment in *S. wallichii. *

Treatments	% fruit set	% seed set
Apomixis	0.00	0.00
Autogamous self-pollination	15 ± 1.14	21.4 ± 2.32
Natural pollination	65.4 ± 6.42	56.6 ± 8.54
Hand self-pollination	38.6 ± 3.18	26.4 ± 3.68
Hand cross-pollination	74.2 ± 5.72	70.8 ± 7.46

## References

[1] Poole RW, Rathcke BJ, Gary Stiles F (1979). Regularity, randomness, and aggregation in flowering phenologies. *Science*.

[2] Ollerton J, Lack AJ (1992). Flowering phenology: an example of relaxation of natural selection?. *Trends in Ecology and Evolution*.

[3] Campbell DR, Motten AF (1985). The mechanism of competition for pollination between two forest herbs. *Ecology*.

[4] Mitchell RJ, Shaw RG, Waser NM (1998). Pollinator selection, quantitative genetics, and predicted evolutionary responses of floral traits in *Penstemon centranthifolius* (Scrophulariaceae). *International Journal of Plant Sciences*.

[5] Kunin WE (1997). Population size and density effects in pollination: pollinator foraging and plant reproductive success in experimental arrays of *Brassica kaber*. *Journal of Ecology*.

[6] Bosch M, Waser NM (1999). Effects of local density on pollination and reproduction in *Delphinium nuttallianum* and *Aconitum columbianum* (Ranunculaceae). *American Journal of Botany*.

[7] Stanton ML, Preston RE (1988). Ecological consequences and phenotypic correlates of petal size variation in wild radish, *Raphanus sativus* (Brassicaceae). *American Journal of Botany*.

[8] Cresswell JE, Galen C (1991). Frequency-dependent selection and adaptive surfaces for floral character combinations: the pollination of *Polemonium viscosum*. *American Naturalist*.

[9] Frankie GW, Opler PA, Bawa KS (1976). Foraging behaviour of solitary bees: implications for outcrossing of a neotropical forest tree species. *Journal of Ecology*.

[10] Stephenson AG (1982). When does outcrossing occur in a mass-flowering plant?. *Evolution*.

[11] Talavera S, Gibbs PE, Herrera J (1993). Reproductive biology of *Cistus ladanifer* (Cistaceae). *Plant Systematics and Evolution*.

[12] Iwaizumi MG, Sakai S (2004). Variation in flower biomass among nearby populations of *Impatiens textori* (Balsaminaceae): effects of population plant densities. *Canadian Journal of Botany*.

[13] Lloyd D, Webb CJ (1986). The avoidance of interference between the presentation of pollen and stigmas in angiosperms, I. Dichogamy. *New Zealand Journal of Botany*.

[14] Snow AA, Spira TP, Simpson R, Klips RA, Lloyd DG, Barrett SCH (1996). The ecology of geitonogamous pollination. *Floral Biology*.

[15] Barrett SCH, Lovett-Dous J, Lovett-Dous L (1988). The evolution, maintenance and loss of self-incompatibility systems. *Plant Reproductive Ecology—Patterns and Strategies*.

[16] Sedgley M, Williams EG, Clarke A, Nox RB (1994). Self-incompatibility in woody horticultural species. *Genetic Control of Self-Incompatibility and Reproductive Development in Flowering Plants*.

[17] Bawa KS, Ng FSP, Bawa KS, Hadley M (1990). Phenology—commentary. *Reprodutive Ecology of Tropical Forest Plants*.

[18] Webber AC, Gottsberger G (1999). Phenological patterns of six *Xylopia (Annonaceae)* species in Central Amazonia. *Phyton*.

[19] Newstrom LE, Frankie GW, Baker HG, Colwell RK, McDade LA, Bawa KS, Hespenheide HA, Hartshorn GS (1994). Diversity of long-term flowering patterns. *La Selva: Ecology and Natural History of a Neotropical Rain Forest*.

[20] Griffiths M (1994). *Index of Garden Plants*.

[21] Tamrakar PR (1992). Management system of natural *Schima/Castanopsis* forest in the middle hills of Nepal. *Banko Jankari*.

[22] Corlett RT (1999). Environmental forestry in Hong Kong: 1871–1997. *Forest Ecology and Management*.

[23] Li-Zhen W, Li ZW (1997). Study on applied effectiveness of biological firebreak network of *Schima*. *Scientia Silvae Sinicae*.

[24] Troup RS (1921). *The Silviculture of Indian Trees*.

[25] Bishop JG, Schemske DW (1998). Variation in flowering phenology and its consequences for lupines colonizing Mount St. Helens. *Ecology*.

[26] McIntosh ME (2002). Flowering phenology and reproductive output in two sister species of *Ferocactus* (Cactaceae). *Plant Ecology*.

[27] Augspurger CK (1983). Phenology, flowering synchrony, and fruit set of six neotropical shrubs. *Biotropica*.

[28] Primack RB (1980). Variation in the phenology of natural populations of montane shrubs in New Zealand. *Journal of Ecology*.

[29] Kearns CA, Inouye DW (1993). *Techniques for Pollination Biologists*.

[30] Ornduff R (1975). Pollen flow in *Lythrum junceum*, a tristylous species. *New Phytologist*.

[31] Molina RT, Rodríguez AM, Palacios IS, López FG (1996). Pollen production in anemophilous trees. *Grana*.

[32] Gross RS, Werner PA (1983). Relationships among flowering phenology, insect visitors, and seed-set of individuals: experimental studies on four co-occurring species of golden rod (*Solidago*: Compositae). *Ecological Monographs*.

[33] Zar J (1999). *Biostatistical Analysis*.

[34] Zapata TR, Arroyo MTK (1978). Plant reproductive ecology of a secondary deciduous tropical forest in Venezuela. *Biotropica*.

[35] Lloyd DG, Schoen DJ (1992). Self- and cross-fertilization in plants. I. Functional dimensions. *International Journal of Plant Sciences*.

[36] Medan D, Bartoloni N (1998). Fecundity effects of dichogamy in an asynchronically flowering population: a genetic model. *Annals of Botany*.

[37] Khanduri VP (2012). Annual variation in floral phenology and pollen production in a 25-year-old plantation of *Tectona grandis*. *Nordic Journal of Botany*.

[38] El-Kassaby YA, Fashler AMK, Sziklai O (1984). Reproductive phenology and its impact on genetically improved seed production in a Douglas-fir seed orchard. *Silvae Genetica*.

[39] Burczyk J, Chalupka W (1997). Flowering and cone production variability and its effect on parental balance in a Scots pine clonal seed orchard. *Annales des Sciences Forestieres*.

[40] Owens JN, Bennett J, L’Hirondelle S (2005). Pollination and cone morphology affect cone and seed production in lodgepole pine seed orchards. *Canadian Journal of Forest Research*.

[41] Lai BS, Funda T, Liewlaksaneeyanawin C (2010). Pollination dynamics in a Douglas-fir seed orchard as revealed by pedigree reconstruction. *Annals of Forest Science*.

[42] Mooney KA, Linhart YB, Snyder MA (2011). Masting in ponderosa pine: comparisons of pollen and seed over space and time. *Oecologia*.

[43] Sork VL, Bramble J, Sexton O (1993). Ecology of mast-fruiting in three species of North American deciduous oaks. *Ecology*.

[44] Kelly D, Sork VL (2002). Mast seeding in perennial plants: why, how, where?. *Annual Review of Ecology and Systematics*.

[45] Sanguinetti J, Kitzberger T (2008). Patterns and mechanisms of masting in the large-seeded southern hemisphere conifer *Araucaria araucana*. *Austral Ecology*.

[46] Caramiello R, Siniscalco C, Mercalli L, Potenza A (1994). The relationship between airborne pollen grains and unusual weather conditions in Turin (Italy) in 1989, 1990 and 1991. *Grana*.

[47] Spano D, Cesaraccio C Phenological stages of natural species and their use as climate indicators.

[48] Opler PA, Frankie GW, Baker HG (1976). Rainfall as a factor in the release, timing, and synchronization of anthesis by tropical trees and shrubs. *Journal of Biogeography*.

[49] Iwasa Y, Satake A (2004). Mechanisms inducing spatially extended synchrony in mast seeding: the role of pollen coupling and environmental fluctuation. *Ecological Research*.

[50] Tapper P-G (1996). Long-term patterns of mast fruiting in fraxinus excelsior. *Ecology*.

[51] Knight TM, Steets JA, Vamosi JC (2005). Pollen limitation of plant reproduction: pattern and process. *Annual Review of Ecology, Evolution, and Systematics*.

[52] Harder LD (1990). Pollen removal by bumble bees and its implications for pollen dispersal. *Ecology*.

[53] Wagenius S, Lyon SP (2010). Reproduction of *Echinacea angustifolia* in fragmented prairie is pollen-limited but not pollinator-limited. *Ecology*.

[54] Allison TD (1990). Pollen production and plant density affect pollination and seed production in *Taxus canadensis*. *Ecology*.

[55] Bawa KS, Bullock SH, Perry DR, Coville RE, Grayum MH (1985). Reproductive biology of tropical lowland rain forest trees. II. Pollination systems. *American Journal of Botany*.

[56] van Vleck LD, Pollak EJ, Oltenacu EA (1987). *Genetics for the Animal Sciences*.

